# Advantages of later motherhood

**DOI:** 10.1007/s00129-017-4124-1

**Published:** 2017-08-29

**Authors:** M. Myrskylä, K. Barclay, A. Goisis

**Affiliations:** 1 Max Planck Institute for Demographic Research, Konrad-Zuse-Straße 1, 18057 Rostock, Germany; 2 London School of Economics and Political Science, London, UK; 3 University of Helsinki, Helsinki, Finland; 4 Stockholm University, Stockholm, Sweden

**Keywords:** Pregnancy, Maternal age, Maternal health, Socioeconomic status, Well-being, Schwangerschaft, Mütterliches Alter, Mütterliche Gesundheit, Sozioökonomischer Status, Wohlbefinden

## Abstract

**Background:**

In high-income countries childbearing has been increasingly postponed since the 1970s and it is crucial to understand the consequences of this demographic shift. The literature has tended to characterize later motherhood as a significant health threat for children and parents.

**Objectives:**

We contribute to this debate by reviewing recent evidence suggesting that an older maternal age can also have positive effects.

**Materials:**

Literature linking the age at parenthood with the sociodemographic characteristics of the parents, with macrolevel interactions, and with subjective well-being.

**Methods:**

Comprehensive review of the existing literature.

**Results:**

Recent studies show that there can also be advantages associated with later motherhood. First, whilst in past older mothers had low levels of education and large families, currently older mothers tend to have higher education and smaller families than their younger peers. Consequently, children born to older mothers in the past tended to have worse outcomes than children born to younger mothers, whilst the opposite is true in recent cohorts. Second, postponement of childbearing means that the child is born at a later date and in a later birth cohort, and may benefit from secular changes in the macroenvironment. Evidence shows that when the positive trends in the macroenvironment are strong they overweigh the negative effects of reproductive ageing. Third, existing studies show that happiness increases around and after childbirth among older mothers, whereas for younger mothers the effect does not exist or is short-lived.

**Conclusion:**

There are important sociodemographic pathways associated with postponement of childbearing which might compensate or even more than compensate for the biological disadvantages associated with reproductive ageing.

## Zusammenfassung

**Hintergrund:**

In Ländern mit hohem Einkommen wird die Realisierung des Kinderwunsches seit den 1970er-Jahren zunehmend verschoben, und es ist wichtig, die Folgen dieses demografischen Wandels zu verstehen. In der Literatur bestand die Tendenz, eine späte Mutterschaft als erhebliches Gesundheitsrisiko für Kinder wie Eltern darzustellen.

**Ziele:**

Wir leisten einen Beitrag zu dieser Debatte, indem wir neuere Daten überprüfen, die darauf hindeuten, dass ein höheres Alter der Mutter auch positive Auswirkungen haben kann.

**Materialien:**

Literatur, die das Alter zu Beginn der Elternschaft mit den soziodemografischen Merkmalen der Eltern, mit Interaktionen auf Makroebene und mit dem subjektiven Wohlbefinden verknüpft.

**Methoden:**

Umfassende Überprüfung der vorhandenen Literatur.

**Ergebnisse:**

Aktuelle Studien zeigen, dass es im Zusammenhang mit einer späteren Mutterschaft auch Vorteile geben kann. Zunächst haben ältere Mütter gegenwärtig tendenziell einen höheren Bildungsabschluss und kleinere Familien als jüngere Mütter, während in der Vergangenheit ältere Mütter ein niedriges Bildungsniveau und große Familien aufwiesen. Bei Kindern älterer Mütter bestand daher in der Vergangenheit ein tendenziell schlechteres Outcome als bei denen jüngerer Mütter, während bei den neuesten Jahrgängen das Gegenteil der Fall ist. Zweitens bedeutet die Verschiebung der Schwangerschaft auf einen späteren Zeitpunkt, dass das Kind in einem späteren Geburtsjahrgang geboren wird und von säkularen Veränderungen in der Makroumgebung profitieren kann. Wenn die positiven Entwicklungen in der Makroumgebung stark genug sind, überwiegen sie nachweislich die negativen Auswirkungen des reproduktiven Alterns. Drittens belegen Studien, dass die Zufriedenheit kurz vor und nach der Geburt des Kindes bei älteren Müttern zunimmt, während dieser Effekt bei jüngeren Müttern überhaupt nicht auftritt oder nur von kurzer Dauer ist.

**Schlussfolgerung:**

Im Zusammenhang mit der verschobenen Realisierung des Kinderwunsches gibt es wichtige soziodemografische Faktoren, welche die biologischen Nachteile des reproduktiven Alterns ausgleichen oder sie sogar mehr als ausgleichen könnten.

In this review we summarise recent research examining how childbearing at older ages affects the health and wellbeing of both the mother and child. Although most research on advanced maternal age focuses on the risks associated with reproductive aging, recent research suggests that older mothers today differ from those in the past, and that there are also benefits to postponing childbearing to older ages. Older mothers today observe better health behaviours during pregnancy, are more socioeconomically advantaged, and seem to be happier after childbearing. Furthermore, the children of older parents in high-income countries have better health and educational outcomes.

## Introduction

Being born to an older parent may represent a significant long-term health risk. A century ago, Alexander Graham Bell suggested that children born to older mothers have the shortest lifespan [1]. According to a recent review, “Parental age has been shown to be a major factor, if not the most important factor, in producing variability in offspring”, including health, longevity and intelligence [2]. As parental ages are now increasing across the developed world, so are concerns about the health and well-being consequences of postponed parenthood. In Germany and the UK, the mean age at first birth exceeded 30 in 2009. In Sweden, the share of children born to mothers over age 40 quadrupled from 1% in 1970 to 4% in 2010; in the same period, the share of children born to mothers under age 20 declined from 8 to 1% [3]. The postponement of childbearing has been attributed to the contraceptive pill, the expansion of career opportunities for women, and increasing economic uncertainty [4–6]. The direct demographic consequences of postponement are many, including decreasing period fertility and changes in the population structure.

Postponement of childbearing may have important health and well-being consequences for children and their parents. For example, the risk of negative birth and childhood outcomes—e. g. Down syndrome, childhood cancer and autism—appear to increase with maternal and/or paternal age [7–9]. Less is known about the potential consequences for offspring adult outcomes, but the existing literature suggests that being born to an older mother or father has severe long-term health consequences, most importantly, old age mortality may be elevated among those who are born to older parents [10–12].

Recent literature, however, also documents important benefits and advantages among those who are born to older parents. In addition, emerging evidence suggests that at least some of the previously accepted negative outcomes that have been associated with advanced maternal age may have been overestimated. Finally, research on the parents suggests that late parenthood may be beneficial when compared to early childbearing.

In this paper we review some of the key advantages that are associated with postponement of childbearing, both for the children and for the parents who postpone childbearing. We discuss first how at the individual level, parenthood at an older age tends to be associated with a socioeconomically and behaviourally beneficial profile that may also be advantageous for child development. Second, we summarise research showing how postponement of childbearing may have beneficial effects on the children through a macrolevel mechanism which emerges because postponement means that the child is born at a later date and to a later birth cohort and may therefore live his or her life in a more advanced society than he or she would, had he or she been born to a younger mother and to an earlier cohort. Finally, we discuss the recent evidence that suggests that older mothers and fathers are better able to enjoy parenthood.

## Benefits associated with older age parenthood

### Older mothers and their sociodemographic and behavioural characteristics

The socioeconomic and behavioural characteristics of the parents are known to be important determinants of birth outcomes and also later child health and well-being [13–15]. In contemporary high-income societies, older parents may be socioeconomically advantaged when compared to younger parents. There is no evidence that older mothers would have enjoyed a socioeconomically advantageous position historically. Much of the evidence on the association between advanced maternal age and compromised outcomes for the offspring, however, come from historical data sets [11, 16–18]. It is therefore possible that some of the disadvantage that is attributed to advanced maternal age could be driven by socioeconomic characteristics of the family to which the child is born, and less by age of the mother.

Myrskylä and Fenelon [10] analysed old-age health, obesity and mortality in the Health and Retirement Study data set that covered individuals born in the 1930s to 1950s in the United States. They documented that those who were born to mothers aged 35 and above had markedly worse overall health, a higher risk of being obese, and a higher risk of death than those that were born to mothers aged 25–34. However, those with older mothers also had mothers with much lower levels of education. For example, among those with maternal age 25–34, 72% had a mother who had at least 8 years of education. Among those with maternal age above 40, only 51% of mothers had at least 8 years of education. Thus in this historical cohort, being born to an older mother was a socioeconomic liability, not an advantage. Consequently, statistical adjustment for lower socioeconomic status among those with older mothers explained up to 30% of the health and mortality disadvantage of those who were born to mothers aged above 35 years.

Goisis et al. [19] analysed four UK birth cohort studies that covered birth cohorts 1958, 1970, 1992 and 2000–2002 and found that over this period, there were strong changes in the sociodemographic and behavioural characteristics of older mothers. In the 1958 and 1970 birth cohorts, socioeconomic status based on occupation and education was highest in the households in which the mothers were aged 25–34 years at birth. However, in the 1992 and 2000–2002 birth cohorts, socioeconomic status was highest in households in which the mothers were aged 35–39 at birth. Smoking while pregnant was high across all maternal age groups in the 1958 and 1970 birth cohorts, but in the 1992 and 2001 birth cohorts, only slightly more than 10% of mothers aged 35 and above smoked during pregnancy, while approximately 40% of mothers aged 20–24 had smoked during pregnancy. These findings suggest that the sociodemographic disadvantage that was historically associated with older maternal age has not only disappeared, but has turned into a potentially important advantage.

In another study, Goisis et al. [20] analysed three UK data sets, covering the birth cohorts 1958, 1970 and 2000–2002, with focus on how child cognitive development is associated with advanced maternal age across these cohorts. The striking finding of this cohort analysis is that while in the 1958 and 1970 birth cohorts children that were born to mothers aged 35 and above scored lower in cognitive development tests at age 11 than those born to mothers aged 25–29, in the 2000–2002 birth cohort this disadvantage had reversed to an advantage: those who were born to mothers aged 35 and above scored higher than those who were born to younger mothers. Goisis et al. document that in the 2000–2002 birth cohorts, those with the oldest mothers also had on average lower birth order and higher family socioeconomic status, both of which are associated with a large number of positive child outcomes [14, 21–24]. These sociodemographic advantages to large extent explained the cognitive ability advantage that was associated with older age motherhood.

### Postponement of parenthood and changing environment

Postponement of childbearing may also have beneficial effects on the children through a macrolevel mechanism. Postponement of childbearing means that a child is born at a later date and to a later birth cohort and may therefore live his or her life in a more advanced society than he or she would had he or she been born to a younger mother and to an earlier cohort. Put another way, from the perspective of an individual parent, a mother or father who postpones childbearing by ten years also gives birth a decade later in calendar years. For example, a woman born in 1950 who gave birth at age 30 gave birth in 1980; if she gave birth at age 40, the birth took place in 1990.

Over the course of the 20^th^ century, and continuing into the 21^st^, there have been steady population-level improvements on several measures that are closely related to individual quality of life. One of the most dramatic of these population-level changes has been the increase in life expectancy. Rapidly decreasing mortality rates amongst children in the first half of the 20^th^ century, and amongst older adults in the second half, have meant that period life expectancy at birth has increased from age 50 in 1900 to over age 80 in 2010 in Sweden, and very similar improvements have been observed across Western Europe, North America and Japan [25–27]. Improvements in cohort life expectancy have been even more dramatic [28]. Other health-related improvements documented over the course of the 20^th^ century include steady increases in population-level cognitive ability [29] and height [30]. Most countries in Western Europe and North America have also witnessed a remarkable expansion of educational opportunity [31]; whereas only a tiny minority went to university in the early part of the 20^th^ century, some countries today see up to 50% of high school graduates making that transition.

Several studies have shown that although advanced maternal and paternal age at childbearing entails increased risks, the population-level improvements over the 20^th^ century described above seem to counterbalance the risks associated with advanced parental age. After taking period improvements into account, children born to older mothers in Sweden have higher cognitive ability [32], are taller, have better grades in high school, and achieve greater educational attainment [33]. Furthermore, children born to older mothers and fathers in Sweden have lower mortality [34]. Recent research suggests that the ability of rapid period improvements to counterbalance the negative effects of reproductive aging are not limited to high-income countries; in low-income countries that have experienced rapid declines in child mortality, children born to mothers at older ages are substantially more likely to survive childhood [35]. The key idea here is that a given child will be relatively better off than if that same individual had been born to the same parents at an earlier point in time.

Overall this recent research suggests that parents who have postponed childbearing in the 20^th^ century have increased the socioeconomic opportunities and health of their children. Any prediction that this association will continue in the future would be based on the assumption that there will continue to be population-level improvements on key measures, such as life expectancy. Although the future is inherently uncertain, the long-term trends in economic growth and declining mortality indicate that future generations will inherit a more prosperous and healthier environment than the one we inhabit today [27].

### Older mothers and maternal well-being

Children may bring with them joy, meaning, and stress and worries. Indeed, the research is very mixed on the topic of whether children overall increase or decrease parental well-being, or happiness: Several studies document that children reduce overall happiness for parents [36, 37] but others find that childbearing increases happiness, at least temporarily [38–40]. It is possible that the results are partially mixed in particular because the impact of children on parental well-being is so domain-specific, for example, children are likely to reduce the hedonic aspects of well-being, by for example reducing leisure time, but may strongly contribute to eudaimonic aspects of well-being that are related to fulfilment, meaning and self-realization. How the needs to hedonic and eudaimonic aspects of well-being balance out may strongly depend on age, and on the socioeconomic resources that are available for parents to alleviate the stress that comes with children.

Research on older mothers suggests that women who postpone childbearing are more ‘ready’ and less stressed by having children [41]. Gregory [41] argues that older mothers are more ready because they have higher status at work which allows greater financial flexibility, options for childcare and more social capital which ease the transition to parenthood. Margolis and Myrskylä [42] also found that the association between parenthood and subjective well-being varies strongly with age: it is negative at young ages (<30), but becomes positive at older ages (50+). However, the study [42] did not include information on the age of mothers at birth, so this study only suggested but did not conclusively show that having children at older ages would be associated with higher subjective well-being.

Myrskylä and Margolis [43] analysed how global subjective well-being, or happiness, changes over time from before having any children to after having one, two or three children using German and British longitudinal data sets. This study differentiated the patterns by age at first birth and found that among young parents (aged 18–22 years at first birth), happiness tends to decline after the birth of the first child. Among middle-aged parents (age 23–34 years at first birth), a temporary increase in happiness was observed around the time of birth, but happiness declined to prebirth levels within a few years after becoming a parent. Among those who experienced their first birth at ages 35 and above, the happiness trajectory around and after the birth is much more positive: happiness is elevated during the year of the birth, and then despite a small drop, remains at levels that are above those observed when childless. The results were highly similar in both the German and British data sets and suggest that the age at which individuals have their first children is a strong predictor of how much they are able to enjoy parenthood.

The vast majority of the studies that we review here pertain to the era that precedes widespread use of prenatal screening, as well as the use of efficient assisted reproductive technologies. These have both changed and will continue to change the landscape of older age motherhood. Prenatal screening is effectively quality control of the developing foetus, and as developmental and chromosomal abnormalities increase with maternal age but can be at least partially aborted based on prenatal examinations—and individuals have decided to use this opportunity—the negative outcomes that have been associated with advanced maternal age are likely to grow even smaller. This, however, may come with the cost of involuntary childlessness. The rise of assisted reproductive technologies is likely to reduce involuntary childlessness, although it is not clear yet to what extent. This is further complicated by potential overconfidence in the insurance function of procedures such as ‘egg freezing’. Moreover, the health implications of the various assisted reproductive technologies on the children and the parents tend to be largely unknown, and uncovering them continues to be an active and important research agenda.

## Conclusions

Since the 1970s, childbearing has been rapidly moving to higher ages and it becoming increasing important to understand the potential negative and positive effects that this transition in childbearing age might have. The epidemiological and medical literature has made great strides in advancing our understanding of ageing of maternal reproductive tract, and how this may influence child outcomes. Most of the evidence on this ageing-based mechanism suggests that older-age motherhood is associated with negative outcomes for the offspring, including lower birth weight, slower cognitive development, and potentially also higher mortality. In this paper we reviewed some of the emerging literature that suggests that older maternal age can also be associated with beneficial outcomes for both the children and the parents. These mechanisms operate mostly through sociodemographic pathways, in contrast to the mechanisms that are related to the physiobiological ageing of the parental reproductive tract.

First, we analysed the literature on the linkage between age at parenthood and socioeconomic and demographic characteristics of the parents. Our survey suggests that still in the early 20^th^ century and perhaps up to mid-20^th^ century older mothers had low levels of education and the children that they had at ages 35 and above already had many older siblings. However, by the end of the 20^th^ century and in the early 21^st^ century, older mothers tend to have higher education than their younger peers, and their children have only few if any older siblings. This means that currently children that are born to older mothers tend to be born to families of high education, and the family resources are not diluted across a large number of siblings. Consequently, the children that are born to older parents are not disadvantaged the same way as the children with older parents used to be. On the contrary, children with older parents tend to have higher cognitive scores than those with younger parents.

Second, we reviewed the literature that focuses on the interactions between individual level childbearing postponement and changing macrolevel socioepidemiological environment. The core idea in this branch of literature is that postponement of childbearing means that the child is born at a later date and to a different birth cohort, and may therefore benefit from secular changes in the macroenvironment. These studies suggest that the positive trends in the macroenvironment may be so strong that they often overweigh any individual level ageing patterns. For example, increases over birth cohorts in cognitive ability and educational attainment, and decreases in mortality have all been so rapid that even moderately small fertility postponement can have an important impact on child outcomes due to the positive trends in these outcomes.

Third, we surveyed the literature on timing of motherhood and subjective well-being of the mothers. This literature suggests that being “ready” is critically important for the ability to enjoy parenthood. Older maternal age is often associated with socioeconomic resources that may help to alleviate the stress that comes with caring for a child. In addition, having a child may reduce the hedonic aspects of well-being, but may contribute to the eudaimonic aspects of well-being, and older mothers may put more weight on the latter than younger mothers. Consequently, the literature finds that happiness increases around and after childbirth among older mothers, whereas for younger mothers the effect does not exist or is very short-lived.

## References

[1] Bell AG (1918). The duration of life and conditions associated with longevity: study of the Hyde geneology.

[2] Liu Y, Zhi M, Li X (2011). Parental age and characteristics of the offspring. Ageing Res Rev.

[3] Human Fertility Database (2017). MPIDR (Germany) and Vienna Institute of Demography (Austria).

[4] Kohler H-P, Billari FC, Ortega JA (2002). The emergence of lowest-low fertility in Europe during the 1990s. Popul Dev Rev.

[5] Schmidt L, Sobotka T, Bentzen JG, Nyboe Andersen A (2011). Demographic and medical consequences of the postponement of parenthood. Hum. Reprod. Update.

[6] Sobotka T (2004). Postponement of childbearing and low fertility in Europe. Doctoral thesis, University of Groningen.

[7] Durkin MS, Maenner MJ, Newschaffer CJ, Lee L-C, Cunniff CM, Daniels JL, Kirby RS, Leavitt L, Miller L, Zahorodny W, Schieve LA (2008). Advanced parental Age and the risk of autism spectrum disorder. Am J Epidemiol.

[8] Johnson KJ, Carozza SE, Chow EJ, Fox EE, Horel S, McLaughlin CC, Mueller BA, Puumala SE, Reynolds P, Von Behren J, Spector LG (2009). Parental age and risk of childhood cancer: a pooled analysis. Epidemiology.

[9] Yip BH, Pawitan Y, Czene K (2006). Parental age and risk of childhood cancers: a population-based cohort study from Sweden. Int J Epidemiol.

[10] Myrskylä M, Fenelon A (2012). Maternal age and offspring adult health: evidence from the Health And Retirement Study. Demography.

[11] Smith KR, Mineau GP, Garibotti G, Kerber R (2009). Effects of childhood and middle adulthood family conditions on later-life mortality: Evidence from the Utah Population Database, 1850–2002. Soc Sci Med.

[12] Jarry V, Gagnon A, Bourbeau R (2013). Maternal age, birth order and other early-life factors: a family-level approach to exploring exceptional survival. Vienna Yearb Popul Res.

[13] Galobardes B, Lynch JW, Davey Smith G (2004). Childhood socioeconomic circumstances and cause-specific mortality in adulthood: systematic review and interpretation. Epidemiol Rev.

[14] Hayward MD, Gorman BK (2004). The long arm of childhood: the influence of early-life social conditions on men’s mortality. Demography.

[15] Strand BH, Kunst A (2007). Childhood socioeconomic position and cause-specific mortality in early adulthood. Am. J. Epidemiol..

[16] Kemkes-Grottenthaler A (2004). Parental effects on offspring longevity – evidence from 17th to 19th century reproductive histories. Ann. Hum. Biol..

[17] Gavrilov LA, Gavrilova NS, Evdokushkina GN, Semyonova VG, Gavrilova AL, Evdokushkina NN, Lapshin EV (1996). Determinants of human longevity: parental age at reproduction and offspring longevity. Longev Rep.

[18] Gavrilov LA, Gavrilova NS (2012). Biodemography of exceptional longevity: early-life and mid-life predictors of human longevity. Biodemography Soc Biol.

[19] Goisis, A., D.C. Schneider, Daniel and M. Myrskylä. 2015. “Secular changes in the association between advanced maternal age and the risk of low birth weight: a cross-cohort comparison in the UK.” MPIDR Working Papers WP-2015-010. 10.1080/00324728.2018.1442584 29582702

[20] Goisis A, Schneider DC, Myrskylä M (2017). The reversing association between advanced maternal age and child cognitive development: evidence from three UK birth cohorts. Int J Epidemiol.

[21] Erikson R, Goldthorpe J (1992). The constant flux: a study of class mobility in industrial societies.

[22] Black SE, Devereux PJ, Salvanes KG (2005). The more the merrier? The effect of family size and birth order on children’s education. Q J Econ.

[23] Barclay K (2015). A within-family analysis of birth order and intelligence using population conscription data on Swedish men. Intelligence.

[24] Barclay K, Kolk M (2015). Birth order and mortality: a population-based cohort study. Demography.

[25] Human Mortality Database (2017). MPIDR (Germany) and University of California: Berkeley (United States).

[26] Wilmoth JR (2000). Demography of longevity: past, present, and future trends. Exp. Gerontol..

[27] Oeppen J, Vaupel JW (2002). Broken limits to life expectancy. Science.

[28] Shkolnikov VM, Jdanov DA, Andreev EM, Vaupel JW (2011). Steep increase in best-practice cohort life expectancy. Popul Dev Rev.

[29] Flynn JR (1987). Massive IQ gains in 14 nations: What IQ tests really measure. Psychol Bull.

[30] Komlos J, Lauderdale BE (2007). The mysterious trend in American heights in the 20th century. Ann. Hum. Biol..

[31] Breen R (2010). Educational expansion and social mobility in the 20th century. Soc Forces.

[32] Myrskylä M, Silventoinen K, Tynelius P, Rasmussen F (2013). Is later better or worse? Advanced parental age and offspring IQ among half a million Swedish men. Am. J. Epidemiol..

[33] Barclay K, Myrskylä M (2016). Advanced maternal age and offspring outcomes: reproductive aging and counterbalancing period trends. Popul Dev Rev.

[34] Barclay, K. and M. Myrskylä. 2016b. “Parental age and offspring mortality: negative effects of reproductive aging are outweighed by secular increases in longevity.” MPIDR Working Paper WP-2016-011. 10.1080/00324728.2017.1411969 29357761

[35] Barclay, K. and M. Myrskylä. 2017. “Fertility postponement could reduce child mortality: evidence from 228 Demographic and Health Surveys covering 77 developing countries.” MPIDR Working Paper WP-2017-005.

[36] Ross CE, Mirowsky J, Goldsteen K (1990). The impact of family on health: The decade in review. J Marriage Fam.

[37] Umberson D, Williams K, Aneshensel CS, Phelan JC (1999). Family status and mental health. Handbook of the sociology of mental health.

[38] Baranowska A, Matysiak A (2012). Does parenthood increase happiness? Evidence for Poland. Vienna Yearb Popul Res.

[39] Clark A, Georgellis Y (2012). Back to baseline in Britain: Adaptation in the BHPS. IZA Discussion Papers.

[40] Clark AE, Diener E, Georgellis Y, Lucas RE (2008). Lags and leads in life satisfaction: a test of the baseline hypothesis. Econ J.

[41] Gregory E (2007). Why women are embracing the new later motherhood.

[42] Margolis R, Myrskylä M (2011). A global perspective on happiness and fertility. Popul Dev Rev.

[43] Myrskylä M, Margolis R (2014). Happiness: Before and after the kids. Demography.

